# Optical Detection of Latent Fingerprints via SBA-15/PhCN Visible-Light-Active Material

**DOI:** 10.1021/acsomega.6c03243

**Published:** 2026-07-18

**Authors:** Nicoletta Rusta, Stefania Porcu, Francesca Picciau, Enrica Tuveri, Riccardo Corpino, Daniele Chiriu, Carla Cannas, Pietro Coli, Roberto Cardia, Pier Carlo Ricci

**Affiliations:** † Department of Physics, 3111 University of Cagliari, S.p. no. 8 Km 0700, 09042 Monserrato, CA, Italy; ‡ Department of Chemical and Geological Sciences, University of Cagliari, 09124 Cagliari, Italy; § Department of Inorganic Chemistry, Charles University, 128 43 Prague 2, Czech Republic; ∥ National Interuniversity Consortium of Materials Science and Technology (INSTM), 50121 Florence, Italy; ⊥ Scientific Investigation Department (RIS) of Cagliari, Via Ludovico Ariosto, 24, 09129 Cagliari, CA, Italy; # Active Label S.r.l spin-off University of Cagliari, Via Africo 5, 09126 Cagliari, Italy

## Abstract

The development of novel materials and techniques for fingerprint detection remains a key challenge in forensic science, particularly for latent prints deposited on complex, multicolored, or low-contrast surfaces. Conventional detection methods often face limitations in sensitivity, selectivity, and environmental compatibility, prompting a search for innovative and sustainable alternatives. In this work, we report the synthesis and application of an SBA-15/PhCN hybrid system as an efficient and eco-friendly material for the visible-light detection of both fresh and latent fingerprints on various substrates. The combination of mesoporous silica SBA-15, characterized by its high surface area and tunable pore architecture, with the photoreactive organic compound phenyl-doped carbon nitride (PhCN) results in a hybrid exhibiting enhanced optical response and strong interaction with fingerprint residues under visible illumination. The hybrid material was synthesized through a facile and reproducible route, thoroughly characterized by spectroscopic and microscopic analyses, and applied via a conventional powder dusting method. Its performance was systematically evaluated on glass, metal, and plastic substrates, demonstrating a high contrast and excellent ridge definition. The SBA-15/PhCN hybrid exhibited strong visible-light activity and high selectivity toward fingerprint residues, confirming its potential as a sustainable, noninvasive, and cost-effective material for advanced forensic investigations.

## Graphical abstract

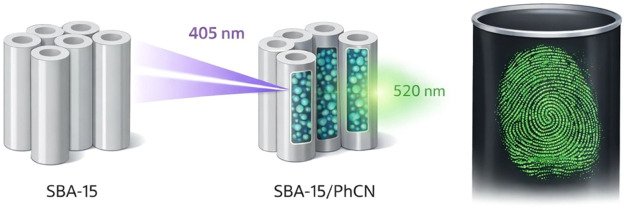

## Introduction

The detection and visualization of latent fingerprints remain central challenges in modern forensic science. Fingerprints provide a fundamental characteristic of personal identification: they are unique to each individual, remain unchanged throughout life, and are universally accepted as reliable evidence in legal and investigative contexts.^1,2^ Despite their importance, obtaining clear and reproducible images of latent fingerprints is often difficult. In practice, prints may be faint, incomplete, or contaminated, and they are frequently deposited on substrates with widely varying textures, porosities, and optical characteristics, ranging from porous paper to smooth metals or plastics, each posing specific difficulties for visualization.^2,3^ Traditional fingerprint development techniques, such as powder dusting, iodine fuming, silver nitrate treatment, DFO (ninhydrin and 1,8-diazafluoren-9-one) reagents, cyanoacrylate fuming, and small particle reagent (SPR) methods, have been utilized for more than a century.^4–8^ Although these approaches are effective in many cases, they also present well-known limitations. Sensitivity can be poor on multicolored or patterned backgrounds, developed prints often degrade over time, and several reagents require toxic or unstable chemicals. In some cases, the methods themselves introduce high autofluorescence or interfere with the original chemical composition of the print, making the analysis of weak or aged impressions particularly challenging. In recent years, nanotechnology has offered new strategies to overcome these limitations. Large surface area, small particle size, and highly tunable surface chemistry are important characteristics of the nanomaterials that enable selective interactions with the complex mixture of organic and inorganic compounds of the fingerprint residues, including amino acids, proteins, fatty acids, and sebaceous lipids.^9^ A wide variety of nanostructures, including metallic nanoparticles (Ag, Au), metal oxides (TiO_2_, ZnO), carbon-based materials, and fluorescent composites, have been explored for this purpose.^10–12^ Emerging revealing techniques, such as micro-X-ray fluorescence (μXRF), chemical imaging, optically stimulated luminescence, synchrotron infrared micro spectroscopy, or energy-dispersive X-ray spectroscopy (EDS), provide new insights into the morphology, composition, and, mainly, the detection of fingerprints.^13,14^ Among them, fluorescent nanomaterials are particularly attractive for the increased contrast between fingerprint ridges and complex backgrounds. Rare-earth-based upconversion nanoparticles, for instance, have been used to minimize background luminescence and improve image clarity.^15–20^ However, their synthesis typically involves toxic heavy metals and the use of high-power excitation beam, which limit their broader applicability. The need therefore remains for luminescent materials that are low-cost, nontoxic, easy to synthesize, and capable of providing strong and selective optical signals in the visible range.^21,22^ Silica-based matrices represent a particularly suitable host for this scope. Mesoporous silica is chemically stable, optically transparent, and biocompatible, with a tunable surface that can be functionalized to improve adhesion to both the aqueous and oily components of fingerprint residues.^12,18,23,24^ Incorporating fluorescent dyes or phosphors within silica frameworks enhances photostability and emission intensity, while maintaining low toxicity. These materials allow for detection under visible light, eliminating the need for ultraviolet excitation or cyanoacrylate fuming and thus offering safer and more sustainable alternatives. To provide a comprehensive comparison between the proposed approach and previously reported fingerprint detection strategies, Table 1 summarizes representative methods based on different materials and detection mechanisms, highlighting their main advantages and limitations.

**Table 1:** Comparison of Representative Fingerprint Detection Approaches Reported in the Literature

**detection approach**	**material/system**	**detection principle/conditions**	**main advantages**	**main limitations**	**refs**
conventional powder dusting	carbon black, magnetic powders, inorganic powders	physical adhesion of powders to fingerprint residues under visible illumination	simple, rapid, inexpensive, widely used in forensic practice	limited selectivity; poor performance on multicolored or complex backgrounds	^4,6^
metal-containing nanoparticles	Ag, Au nanoparticles and nanostructured particles	interaction between nanoparticles and fingerprint residues; optical visualization	high sensitivity and improved ridge contrast	possible aggregation, expensive synthesis, environmental concerns related to metals	^10,11^
ZnO–SiO_2_ nanopowders	ZnO-SiO_2_ hybrid nanoparticles	adsorption-based fingerprint development on nonporous surfaces	good adhesion and enhanced visualization capability	limited optical response without additional functionalization	^12^
fluorescent nanomaterials	carbon dots, quantum dots, fluorescent nanoparticles	fluorescence imaging under UV/visible excitation	high contrast, background reduction, improved sensitivity	some materials require complex synthesis and may involve toxic components	^15,21,22^
organic fluorescent probes	small-molecule fluorescent dyes and functional probes	selective interaction with fingerprint components followed by fluorescence activation	high sensitivity, rapid visualization, good ridge-level imaging	probe synthesis and optimization required	^17,31,32^
fluorescent silica nanoparticles	functionalized silica-based fluorescent nanoparticles	fluorescence enhancement through silica-supported fluorophores	chemical stability, tunable surface chemistry, low aggregation	requires additional functionalization to introduce optical activity	^18,19^
mesoporous silica-based materials	functionalized mesoporous silica/SBA-15 systems	adsorption of fingerprint residues combined with optical detection	high surface area, ordered pores, tunable surface properties	silica alone shows limited luminescence activity	^23,24^
optically stimulated luminescence materials	luminescent inorganic materials	optical stimulation and luminescence readout	high sensitivity and detection of hidden fingerprints	requires specialized instrumentation	^14^
**SBA-15/PhCN hybrid (this work)**	**phenyl-doped carbon nitride incorporated into mesoporous SBA-15**	**visible-light fluorescence excitation (405 nm)**	**visible-light activation**, **high surface area**, **improved adhesion**, **reduced background interference**, **sustainable composition**	**further validation required with larger forensic data sets and more complex environmental conditions**	**this work**

Compared with previously reported fingerprint detection materials, the SBA-15/PhCN hybrid combines the advantages of a mesoporous inorganic host with the optical properties of a visible-light-active organic semiconductor. The SBA-15 matrix provides high surface area and ordered porosity, favoring interaction with fingerprint residues, while PhCN introduces strong visible-light absorption and emission properties. This combination enables fingerprint visualization under visible excitation, reducing the limitations associated with UV illumination and highly fluorescent backgrounds. On these bases, the present work investigates a hybrid system composed of phenyl-doped carbon nitride (PhCN) incorporated into mesoporous silica SBA-15. Compared to previously reported silica-based or fluorescent fingerprint powders, the present approach exploits a mesoporous host–guest architecture based on SBA-15 and phenyl carbon nitride, allowing both visible-light activation and enhanced interaction with fingerprint residues. In particular, the system can be excited under visible light (∼405 nm), minimizing background autofluorescence commonly associated with UV-based detection methods, while the ordered mesoporous structure promotes improved adhesion to latent fingerprint residues. The combination of these features provides enhanced optical contrast on complex or low-contrast substrates. A common difficulty with silica–phosphor composites lies in embedding luminescent species without compromising their optical performance. Carbon nitride polymers, however, can be obtained from the thermal synthesis of nitrogen-rich precursors that determine the final triazine- or heptazine-based networks. Among these, phenyl-doped carbon nitride displays several features particularly suited to forensic applications: it can be synthesized at moderate temperatures (400 °C from 2,4-diamino-6-phenyl-1,3,5-triazine as precursor), exhibits high quantum efficiency (above 40%), and absorbs efficiently in the visible range (up to 450 nm).^25^ The last property is crucial for practical fingerprint detection, as visible excitation minimizes substrate autofluorescence and reduces operational hazards compared to UV-based techniques. The integration of PhCN within the SBA-15 framework combines the strong optical response of the polymer with the high surface area and porosity of the silica matrix, enhancing its ability to interact with fingerprint residues. The resulting SBA-15/PhCN hybrid was synthesized through a simple and reproducible procedure, characterized by spectroscopic and microscopic techniques, and applied via the powder dusting method for both fresh and latent fingerprint detection. The material shows clear ridge contrast and stable fluorescence across various substrates, highlighting its promise as a sustainable, noninvasive, and efficient material for next-generation forensic fingerprint detection.

## Materials and Methods

Pluronic P123 (average number-average molecular weight ≈ 5800, Sigma-Aldrich), HCl (37%, VWR Chemicals, Radnor, PA, USA), and TEOS (98%, Acros Organics) were used in the synthesis of the SBA-15 support. 2,4-Diamino-6-phenyl-1,3,5-triazine (C_6_H_5_(C_3_N_3_)­(NH_2_)_2_, Ph-Triazine,99%, Sigma-Aldrich) was used for the synthesis of PhCN. Absolute ethanol (C_2_H_5_OH, 96%, Alfa Aesar) was used as solvent for the synthesis of the SBA-15/PhCN hybrid system. All chemicals were used as received without further purification.

### Synthesis of the SBA-15 Support

The synthesis of the SBA-15 support was performed according to the procedure described in.^26^ Typically, 4 g of Pluronic P123 was dissolved in 120 g of HCl (2 M) and 30 g of bidistilled water in an Erlenmeyer flask. The mixture was stirred at 600 rpm at 35 °C in a water bath for 24 h. Subsequently, the stirring speed was reduced to 100 rpm and maintained overnight. Afterward, 9 g of TEOS was added dropwise, and the mixture was stirred for an additional 24 h at 35 °C, and the resulting suspension was transferred to a sealed Teflon-lined autoclave and heated at 100 °C under static conditions for an additional 24 h. The final product was filtered, washed with warm distilled water (75–80 °C), and dried at 35 °C for 24 h. Finally, the obtained powder was calcined at 550 °C for 6 h with a heating rate of 5 °C min^–1^.

### Synthesis of PhCN

Phenyl-modified carbon nitride (PhCN) was prepared by heating 1 g of 2,4-diamino-6-phenyl-1,3,5-triazine powder (Ph-Triazine) in a closed flask in a muffle furnace at 400 °C for 2 h with a heating rate of 10 °C/min and a cooling rate of 5 °C/min.^25^

### Synthesis of SBA-15/PhCN Hybrid System

The SBA-15/PhCN hybrid system was synthesized by mixing the precursors in ethanol under continuous stirring for 2 h to ensure proper interaction. The resulting mixture was then transferred to an oven and subjected to heat treatment at 400 °C for 2 h. The heating process was carried out with a ramp rate of 10 °C per minute until the target temperature was reached. After cooling to room temperature, the resulting brownish powder was collected and finely ground using a mortar to obtain a homogeneous powder. The relative amount of precursor (15 wt % SBA-15 and 85 wt % triazine precursor) was selected to promote the formation of the PhCN phase while preserving the ordered mesoporous structure of SBA-15. The synthesis conditions were carefully controlled to ensure consistency of the obtained material across different preparations. The process for the synthesis of the hybrid system is reported in Scheme 1.

**Scheme 1:**
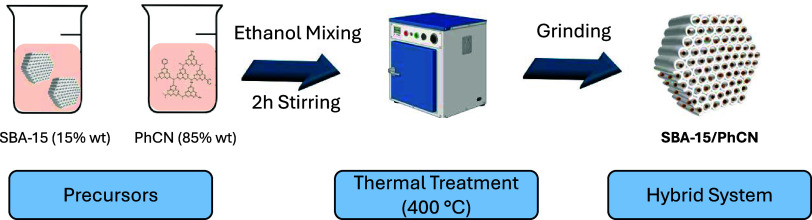
Synthetic Process of the SBA-15/PhCN Hybrid System

### Characterization Techniques

A wide-angle X-ray diffraction (WA-XRD) pattern was acquired in the 2θ range of 10–80° using a PANalytical X’Pert Pro (Malvern PANalytical, Malvern, UK) equipped with a Cu Kα source (1.5418 Å). Low-angle X-ray diffraction (LA-XRD) patterns were acquired in the 2θ range of 0.7–3° using a Seifert X3000 instrument (Seifert, Radevormwald, Germany) equipped with a Cu Kα source. The hexagonal lattice parameter of the mesostructured samples was calculated using the equation

$$a_{0}=2d_{100}/\surd 3$$

Nitrogen physisorption isotherms were acquired at −196 °C using the Micromeritics ASAP 2020 system. All samples were preheated under vacuum at 250 °C (heating rate, 1 °C min^–1^) for 12 h.

A JEOL JEM 1400-PLUS microscope operating at 120 kV was used to obtain the transmission electron microscopy (TEM) micrographs. The samples’ powders were dispersed in *n*-octane, sonicated, and the resulting suspensions were dropped onto 200 mesh carbon-coated copper TEM grids.

### Application of SBA-15/PhCN for Detection of Fingerprints

The SBA-15/PhCN system was applied to study latent fingerprints. Fluorescence mapping studies were conducted by using a 405 nm laser as the excitation source, providing detailed information on the spatial distribution of the fluorescent marker. Additionally, fluorescence imaging studies were performed to further evaluate the sensitivity and efficiency of the system in highlighting fingerprint features. To test reproducibility, fingerprint detection on different substrates was evaluated in triplicate after material deposition without any aging process.

## Results and Discussion

The SBA-15/PhCN system was first characterized to study its optical and structural properties and then applied to detect fresh and latent fingerprints.

The wide-angle XRD graph (Figure 1a) shows the diffraction patterns for pure PhCN and the hybrid material SBA-15/PhCN. The pattern of pure PhCN exhibits a well-defined peak at 27.7°, which is typical of its crystalline structure,^25^ assigned to the distance among planes, due to the (001) reflection. The broad peak at about 15° is associated with the separation distance of heptazine chains and related to the reflection from the (210) plane. When incorporated into the SBA-15 matrix, the main PhCN diffraction peak slightly shifts from 27.7 to 27.4°. According to Bragg’s law, this shift toward lower 2θ values corresponds to a slight increase in the interplanar spacing. This variation suggests a modification of the packing arrangement of PhCN, likely induced by its interaction with the SBA-15 framework and by the confined environment within the mesoporous structure.

**Figure 1:**
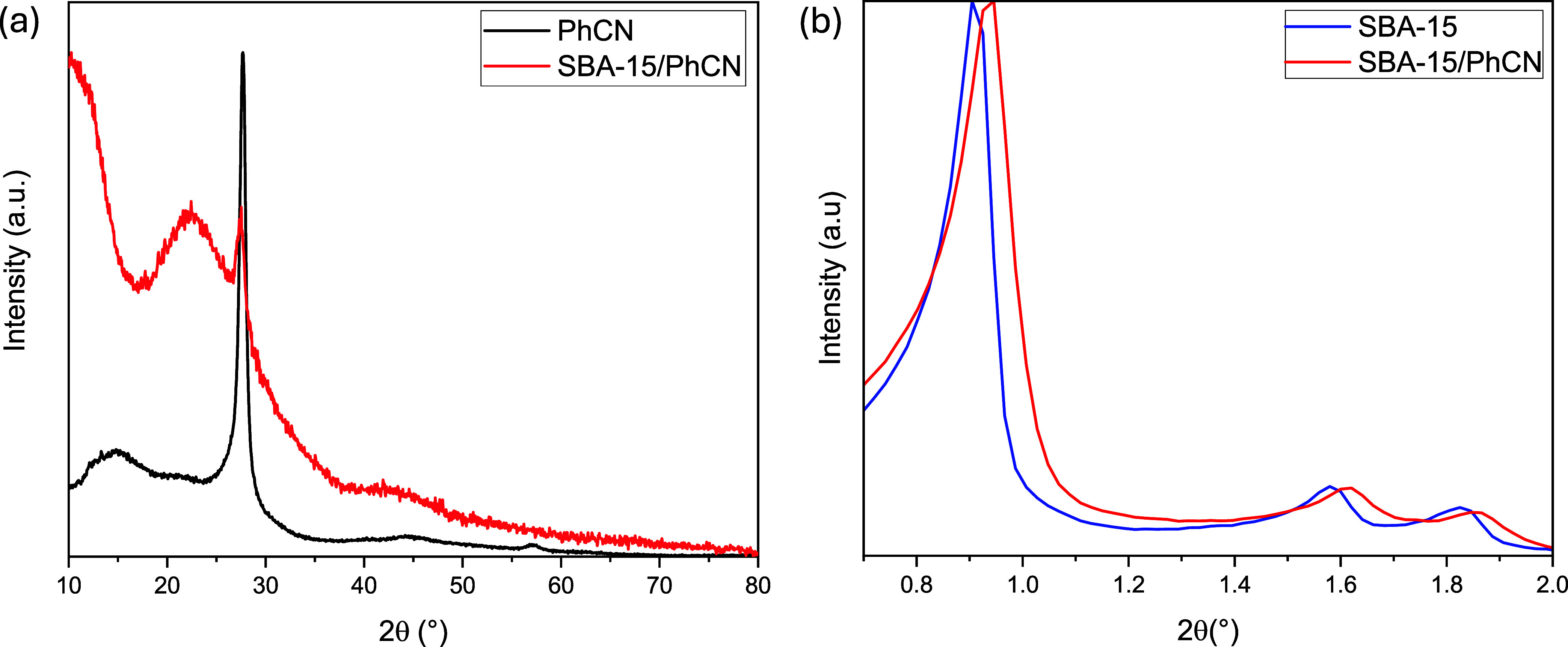
Wide-angle (a) and low-angle (b) X-ray diffraction patterns of PhCN (black) and SBA-15/PhCN hybrid system (red).

The low-angle XRD measurements for the SBA-15 sample and the hybrid material SBA-15/PhCN give information on the mesoporous organization and the effect of PhCN incorporation (Figure 1b). The pattern for SBA-15 displays well-defined peaks, typical of ordered mesoporous structures in the low-angle range (0.8–2.0 Å). These peaks correspond to the (100) reflections of crystallographic planes associated with the hexagonal symmetry of the structure (*P*6*mm*), and their intensity and position confirm the highly ordered nature of the mesoporous structure.^27^

In the case of SBA-15/PhCN, the main peaks of the SBA-15 structure are still present, but a reduction in intensity and a slight shift toward lower angle values are observed. The reduction in intensity is attributed to the filling of the pores with PhCN, while the slight shift suggests a potential modification of the mesoporous structure’s periodicity, probably caused by stresses introduced by the PhCN loading. This interpretation is further corroborated by the lattice parameter analysis, which evidences a decrease after PhCN incorporation, in line with a slight contraction of the mesoporous framework (see Table 2).

**Table 2:** Parameters for the Calculation of Lattice Parameters with Cu Kα Source, Lattice Parameters *a*_0_, Mean Pore Diameter *D*_p_ Obtained from BJH Analysis, and the Corresponding Wall Thickness (T_w_)

	**2θ (°)**	**θ (°)**	**θ (rad)**	***d***_**100**_ **(nm)**	***a***_**0**_ **(nm)**	***D***_**p**_ **BJH (nm)**	**T_w_ (nm)**
**SBA-15**	0.9046	0.4523	0.007894	9.76	11.28	5.59	5.68
**SBA-15/PhCN**	0.9455	0.4727	0.008251	9.33	10.78	5.03	5.75

From the position of the low-angle diffraction peaks, it was possible to calculate the interplanar spacing (*d*-spacing) using Bragg’s equation:

nλ=2dsin⁡θ

where *n* is the order of diffraction (typically *n* = 1), λ is the X-ray wavelength, *d* is the interplanar spacing, and θ is the Bragg angle corresponding to the observed peak.

From the *d*-spacing values, it was then possible to calculate the lattice parameter (*a*_0_) according to the hexagonal symmetry (Table 2):

$$a_{0}=2d_{100}/\surd 3$$

To verify the filling of the pores of SBA-15 with PhCN, N_2_ physisorption measurements have been performed. The adsorption isotherms shown in Figure 2a correspond to type IVb,^28^ which is characteristic of mesoporous materials. For SBA-15, the isotherm shows a well-defined capillary condensation adsorption branch with an H1-type hysteresis loop, suggesting the presence of ordered mesopores. After modification with PhCN, a reduction in adsorption volume is observed, indicating partial pore filling with PhCN. This decrease in surface area and pore volume is consistent with effective encapsulation. Further insight is provided by the desorption branch of the capillary condensation, which exhibits a double concavity. This feature suggests partial obstruction of the mesopores, leading to the formation of inkbottle mesopores (mesopores with a narrow opening) and resulting in slower pore emptying during desorption, thus confirming the presence of PhCN within the pores. Similar desorption behavior has been consistently reported in studies on metal oxide-loaded mesoporous materials,^29^ further validating this interpretation. The pore size distribution, displayed in Figure 2b and calculated using the BJH method, further supports these observations. SBA-15 exhibits a narrow distribution with characteristic mesoporous dimensions (∼6–8 nm). Following modification, the BJH plot shows a broad bimodal distribution, with a peak at 6.2 nm, close to the original pore size of SBA-15, and another peak at approximately 4 nm, supporting the hypothesis of partial pore obstruction. Specifically, the peak at 6.2 nm corresponds to unoccupied pores, while the peak at 4 nm is attributed to inkbottle mesopores created by the incorporation of PhCN.

**Figure 2:**
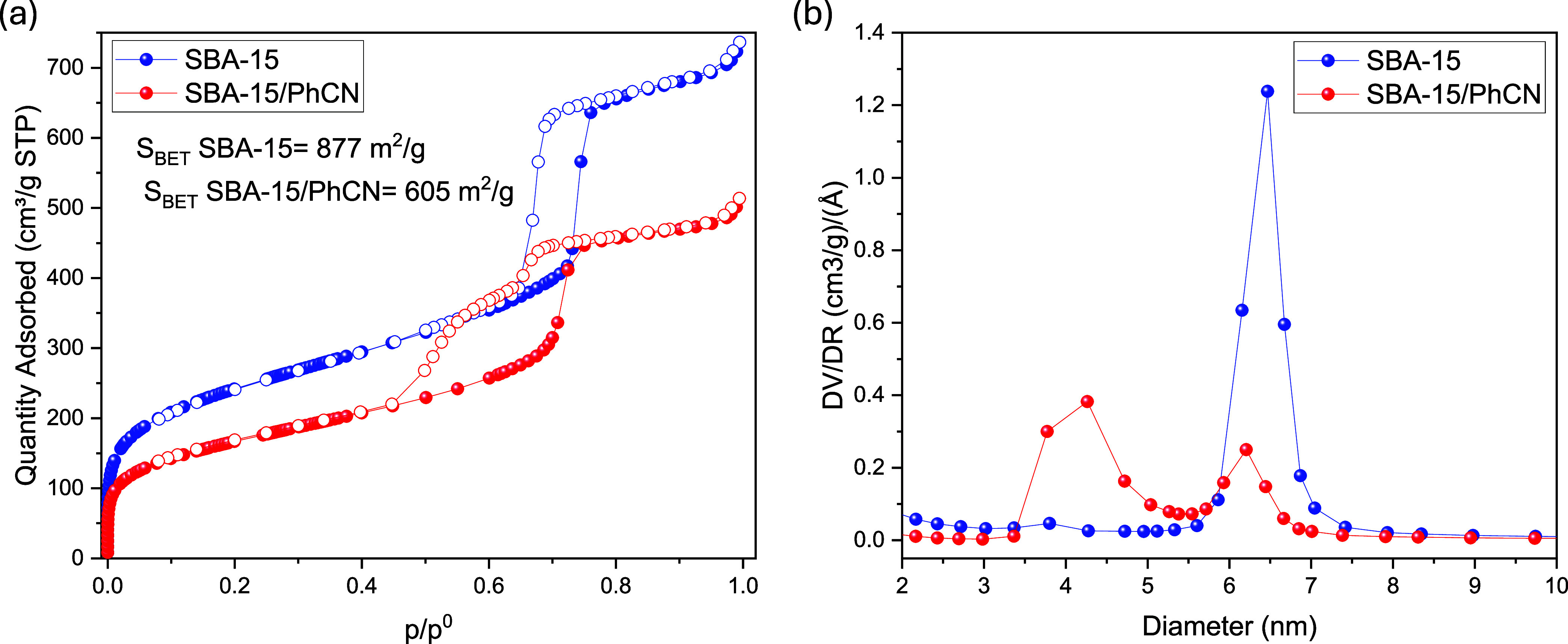
N_2_ physisorption isotherms (a) and pore size distribution (b) of SBA-15 and SBA-15/PhCN hybrid system.

By combining the data obtained from the calculation of the lattice parameters with the pore diameter, it was possible to obtain the wall thickness with the following equation:

$$T_{w}=a_{0}-d_{p}$$

The wall thickness for SBA-15 was 5.68, while that for the hybrid system was 5.75. Increased values of this parameter indicate filling of the pores of SBA-15 with PhCN.

The TEM micrographs (Figure 3) of both bare SBA-15 and the PhCN-modified system show a highly ordered mesoporous structure with hexagonally arranged parallel channels, consistent with previous reports on the SBA-15 materials. No morphological changes are observed after PhCN incorporation, indicating that the mesostructure of SBA-15 is preserved, in agreement with the observations from low-angle XRD and N_2_ physisorption measurements described above.

**Figure 3:**
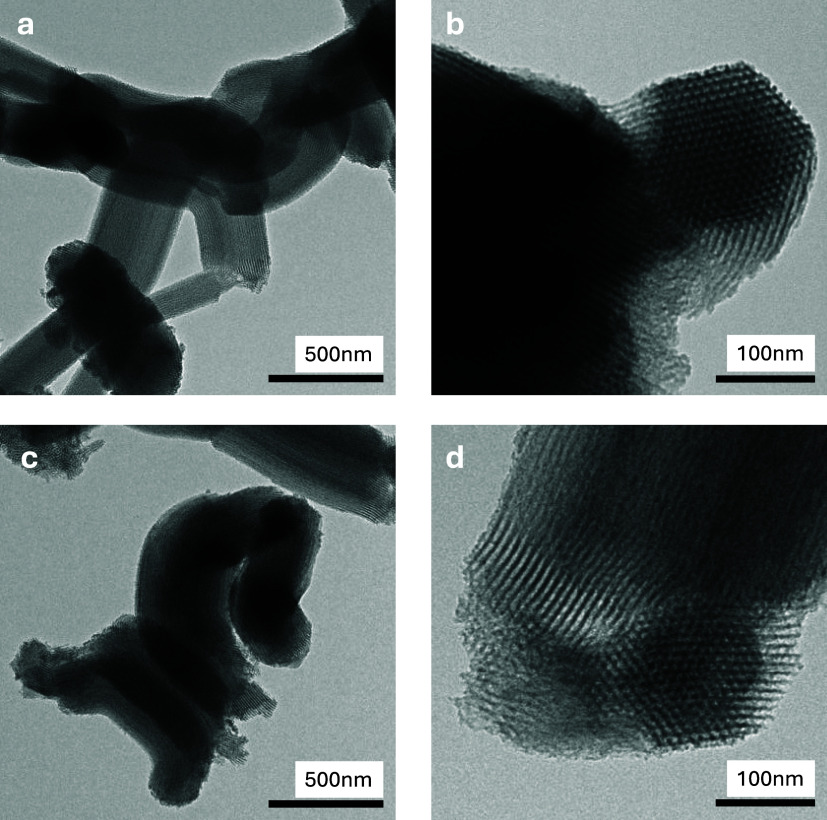
TEM micrographs of SBA-15 (a, b) and SBA-15/PhCN hybrid system (c, d).

To investigate the optical behavior of both the pure components and the hybrid material SBA-15/PhCN, diffuse reflectance spectroscopy (DRS) was employed to record the corresponding absorption spectra (Figure 4). The SiO_2_ matrix exhibits absorption only in the deep ultraviolet region,^30^ whereas the SBA-15/PhCN hybrid displays the characteristic absorption edge associated with phenyl-doped carbon nitride polymers. The absorption onset occurs within the visible region with a maximum around 430 nm, in good agreement with the spectrum of pure PhCN. This observation provides further confirmation of the successful formation of the polymeric phase from the precursor 6-phenyl-1,3,5-triazine-2,4-diamine; indeed, the triazine precursor itself does not show any absorption features in the visible range, exhibiting instead a strong edge in the ultraviolet region at approximately 350 nm.^25^

**Figure 4:**
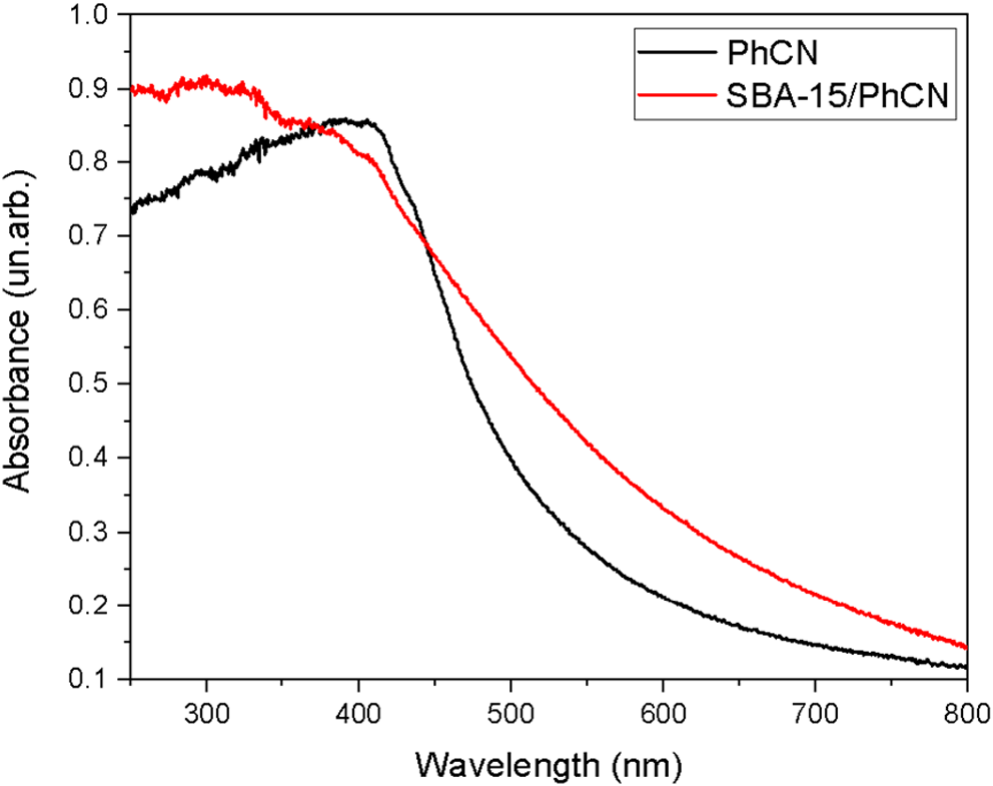
UV–vis absorption spectra of PhCN (black) and SBA-15/PhCN hybrid system (red).

The three-dimensional photoluminescence excitation (PLE) maps are consistent with the absorption results (Figure 5). A broad emission band is observed across the visible region, with a maximum centered at about 520 nm and excited most efficiently around 400 nm. This luminescence feature is present in both the pure PhCN and the SBA-15/PhCN hybrid, confirming that the photophysical properties of the polymer are preserved upon incorporation into the silica framework.

**Figure 5:**
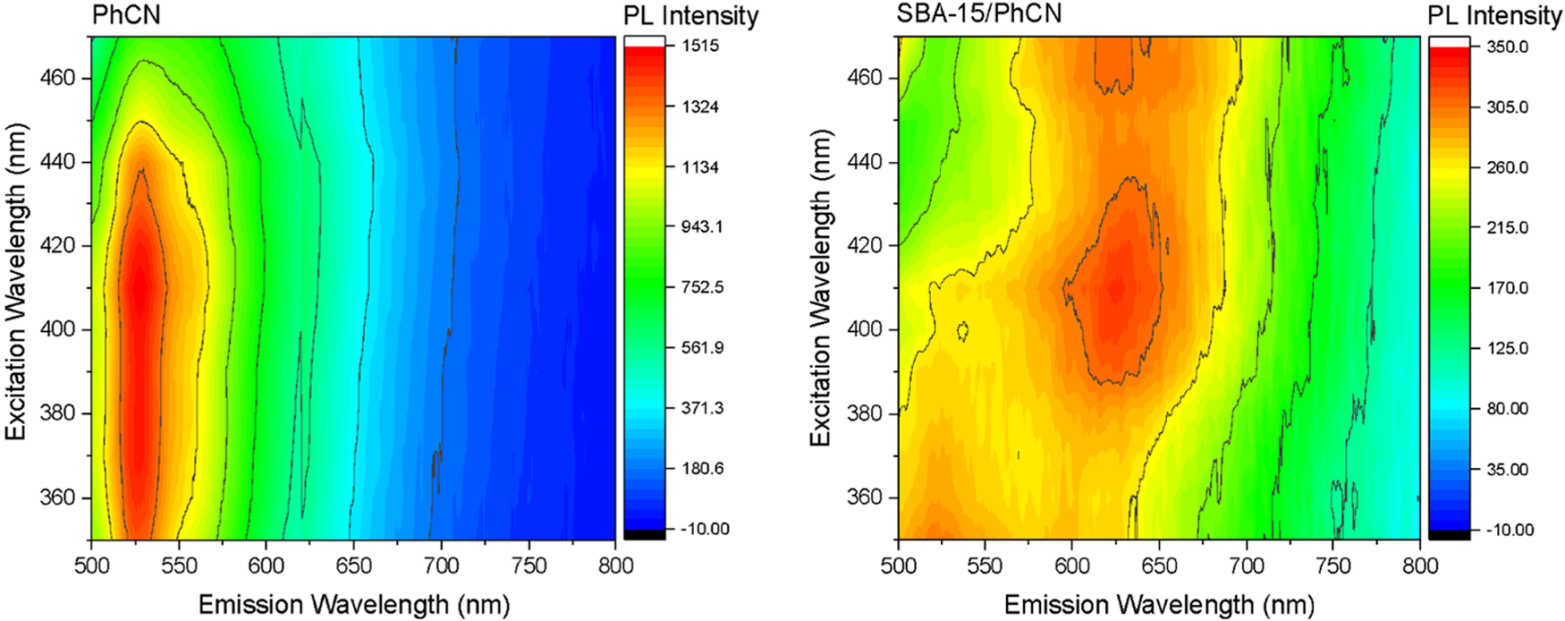
Photoluminescence excitation maps of PhCN and SBA-15/PhCN hybrid system.

However, the steady-state photoluminescence intensity (under 405 nm excitation, i.e., in the excitation maximum of the PhCN and of the SBA-15/PhCN hybrid) is reduced in the hybrid, primarily due to the lower concentration of the emissive phase within the mesoporous matrix. A slight red shift of the emission maximum is also observed, which can be attributed to modifications in molecular packing and to the spatial confinement of the polymeric domains inside the silica pores (Figure 6).^25^ A comprehensive photophysical characterization of PhCN, including quantum yield, fluorescence lifetime, and photostability, has been reported in our previous work. As reported therein, the absolute quantum yield of the material is around 60%, and the time-resolved measurements showed a three-component decay profile with τ = 740, 151, and 36 ns.^25^ In the present study, the optical investigation is specifically focused on evaluating the suitability of the SBA-15/PhCN hybrid for imaging applications; therefore, steady-state photoluminescence and excitation mapping were considered sufficient to assess the visible-light response relevant for fingerprint detection. Since the silica matrix acts essentially as an inert template/support and does not significantly interact with the emissive PhCN system, the photophysical parameters reported in our previous work are not expected to be substantially affected upon incorporation into SBA-15.

**Figure 6:**
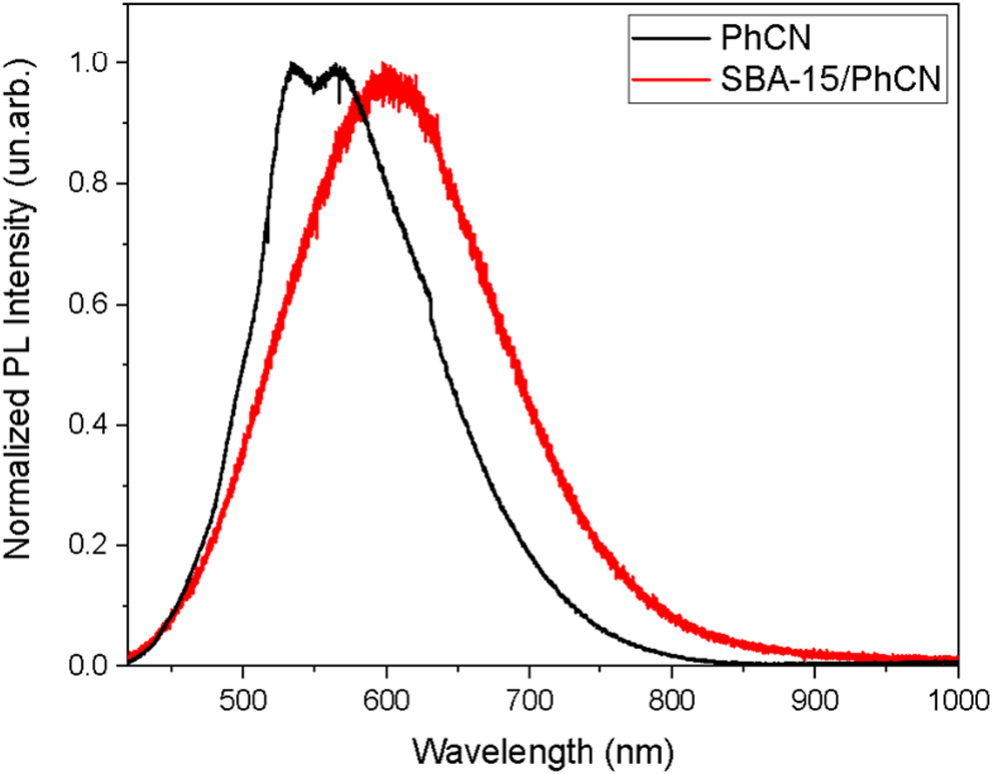
Steady-state luminescence (λ_exc_ = 405 nm) of PhCN (black) and SBA-15/PhCN (red).

It is worth noting that SBA-15 does not exhibit significant absorption or emission in the visible region, as generally reported for mesoporous silica materials and confirmed in our previous work.^26^ Therefore, the optical response observed for the hybrid material can be attributed to the presence of PhCN. Moreover, the slight red shift of the emission and the preservation of the photoluminescence features suggest that the polymer experiences a modified local environment, consistent with spatial confinement. When considered together with the changes in surface area, pore volume, pore size distribution, and low-angle XRD patterns, these results support the incorporation of PhCN within the mesoporous channels rather than simple external deposition.

The optical properties of the hybrid material were subsequently utilized for fingerprint detection experiments. Fluorescence maps were recorded over a 50 mm × 50 mm area under 405 nm excitation (step of 0.5 mm for 10,000 spectra total, Figure 7a). Figure 7b reports the zoom-in of a 2.5 mm × 2.5 mm zone. The color map is obtained from the luminescence acquired at each point, and it corresponds to the integrated emission of the PL between 500 and 800 nm, in accordance with the static PL reported in Figure 7. The fluorescence maps reveal a clear contrast between ridge and background regions, with emission selectively localized on fingerprint residues, enabling clear visualization of ridge patterns.

**Figure 7:**
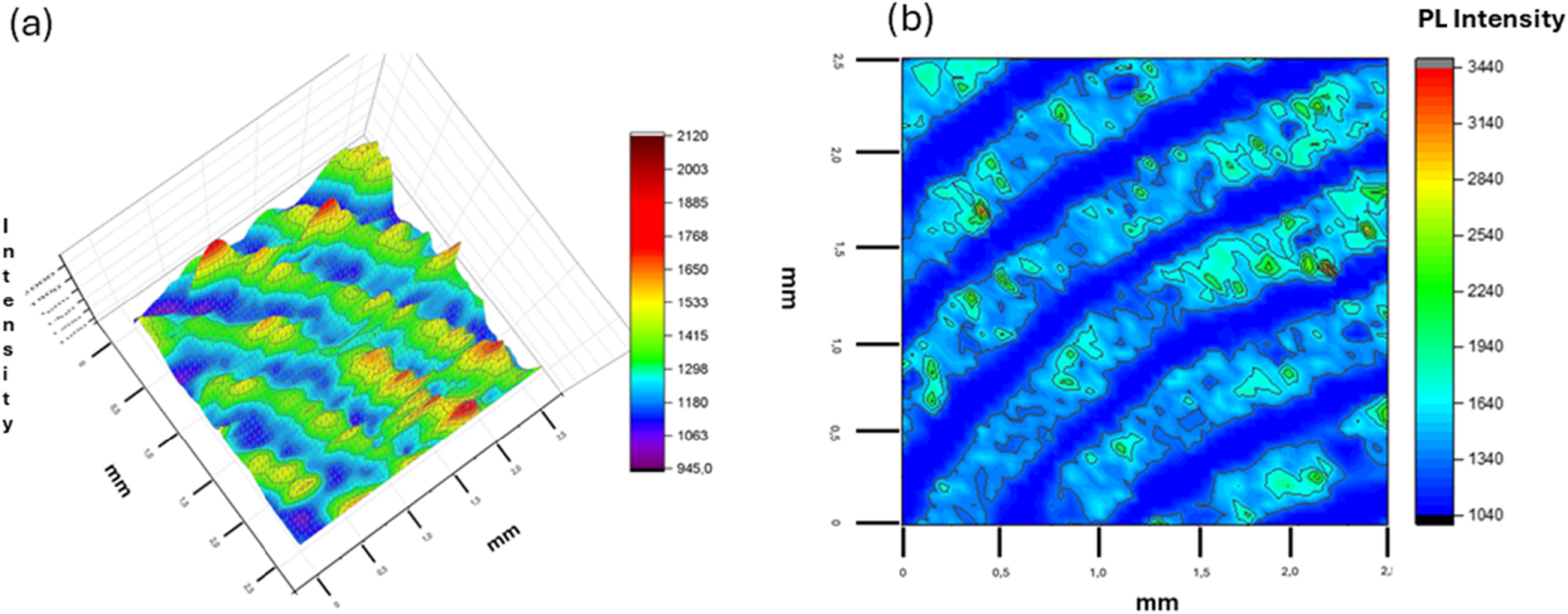
Fluorescence maps recorded over a 50 mm × 50 mm area under 405 nm excitation (a) and zoom-in of a 2.5 mm × 2.5 mm zone (b).

The resulting images clearly reveal the ridge patterns of fingerprints through bright, well-defined regions, corresponding to areas where the polymer preferentially accumulated on the fingerprint residues. In these regions, the fluorescence spectrum characteristic of PhCN is observed, whereas in the surrounding residue-free areas, no emission signal is detected. Figure 8 provides a detailed 3D representation of the same region, further highlighting the spatial distribution of the luminescent signal.

**Figure 8:**
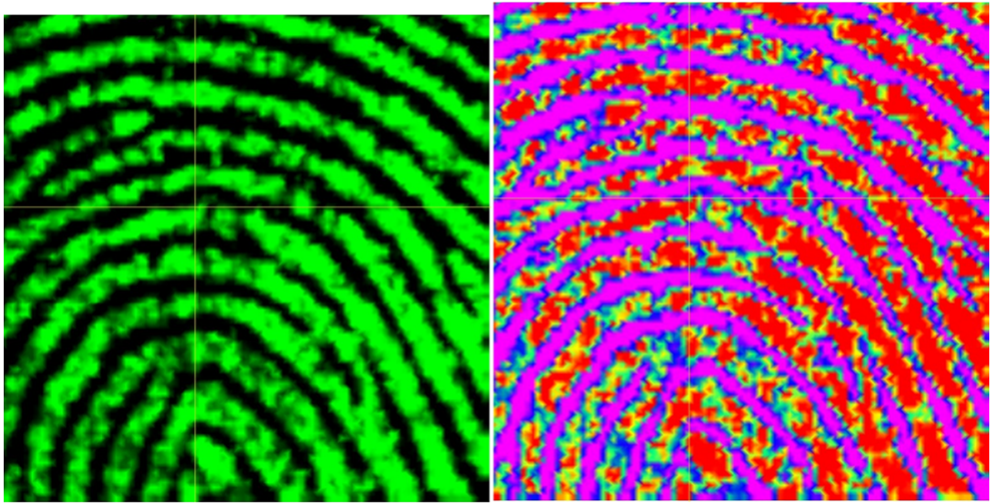
3D fluorescence maps of latent fingerprints.

Latent fingerprints were deposited under controlled conditions: after carefully washing the hand with soap and water, the donor gently massaged the forehead to collect the natural secretions of the skin, then pressed the fingertip on the chosen substrate. Latent fingerprints were placed in depletion to simulate the variability of the presence of secretions. A silicone swab replicating a human footprint, which was soaked in an artificial sweat solution, was used as a comparison impression. The contact time of the swab with the sample solution was 1, 3, and 5 s to simulate the variability of the concentration of the solution on the test swab. The test fingerprint and the real fingerprint are in agreement. Samples were prepared on different surfaces, including glass, plastic, and aluminum, to assess the versatility of the SBA-15/PhCN material. As illustrated in Figure 9, a direct comparison (carried out under identical experimental conditions, including the same deposition procedure and excitation parameters) between pure PhCN and the SBA-15/PhCN hybrid demonstrates the superior adhesion and definition achieved with the hybrid material. The preferential adhesion of the hybrid material to fingerprint residues can be attributed to the combined effect of the high surface area and mesoporous structure of SBA-15, which promotes interactions with both hydrophilic and lipophilic components of the residues, and the presence of the organic PhCN phase, which enhances affinity toward organic compounds. This combined effect contributes to the improved selectivity observed for the hybrid material. In addition, the detection of aged fingerprints, up to 7 days old, confirmed the material’s stability and effectiveness over time, supporting its potential use for practical forensic applications, demonstrating the feasibility of detecting aged fingerprints within a short-term time scale.

**Figure 9:**
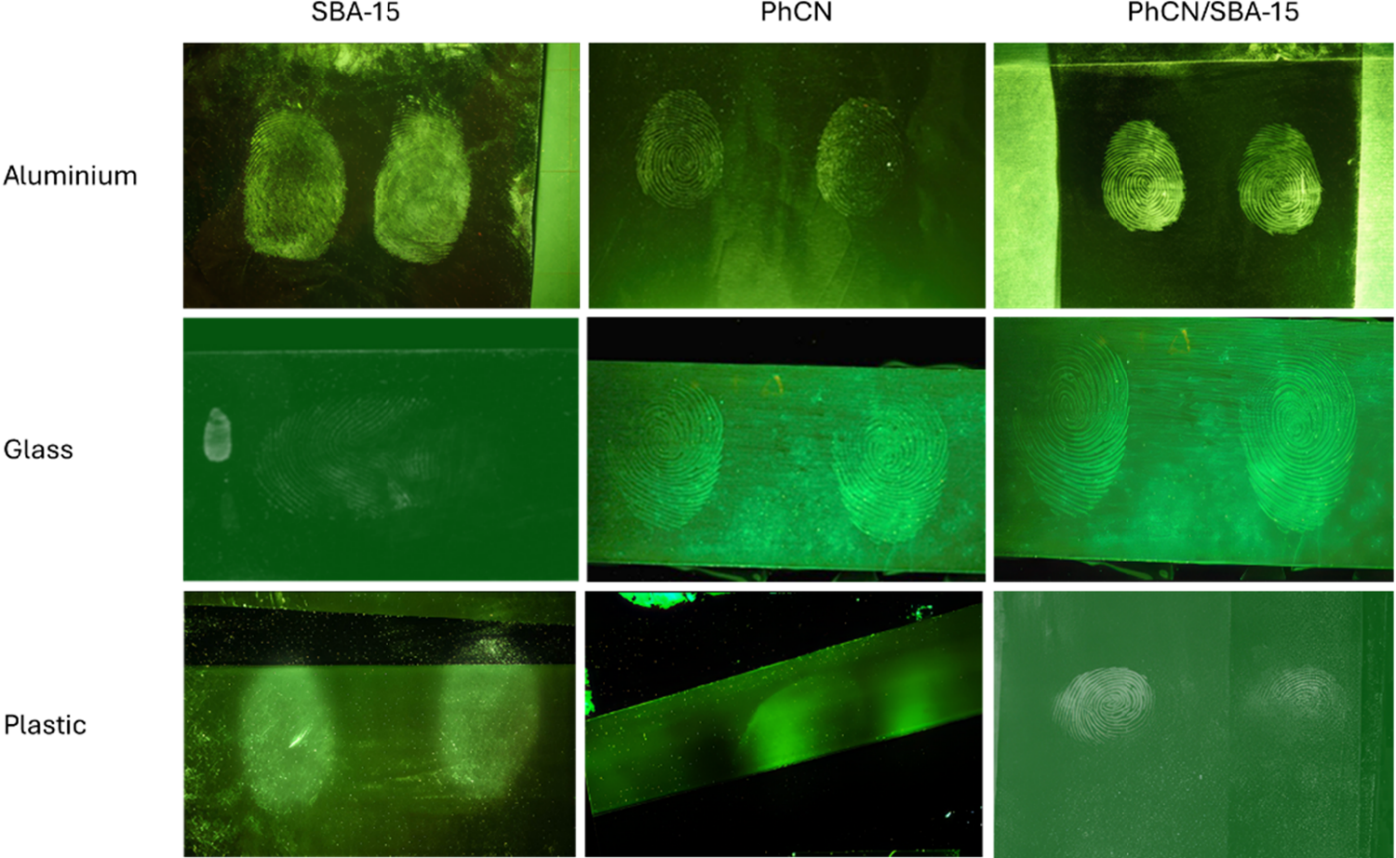
Fingerprints detection over multiple surfaces.

Finally, an application in a real context, represented by a commercial aluminum soft drink can, is reported in Figure 10. As can be observed, the fingerprint can be easily distinguished, including the specific ridge patterns (loops, whorls, and arches).

**Figure 10:**
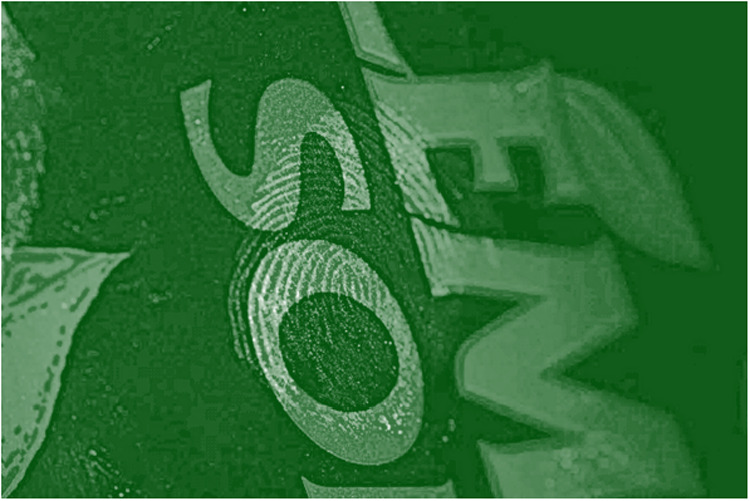
Fingerprint detection on commercial aluminum soft drink can.

## Conclusions

In this work, an eco-friendly and visible-light-active hybrid material based on mesoporous silica SBA-15 and phenyl-doped carbon nitride (PhCN) was successfully synthesized and applied for the detection of both fresh and latent fingerprints. Structural and optical characterizations, including XRD and N_2_ physisorption, confirmed the effective incorporation of PhCN within the SBA-15 mesopores while maintaining the ordered hexagonal framework. From an applied perspective, the integration of PhCN into the silica matrix facilitates a “host–guest” interaction, where the spatial confinement of the polymer domains induces a slight red shift in the emission spectra. The system’s strong absorption edge at 405 nm allows for high-contrast imaging under visible-light excitation. This significantly reduces the background autofluorescence typically encountered with UV-based techniques and eliminates the operational hazards associated with shorter wavelengths. The SBA-15/PhCN hybrid demonstrated superior adhesion and selectivity toward fingerprint residues compared to the pure organic polymer, providing exceptional ridge definition, including loops, whorls, and arches, on challenging nonporous substrates such as glass, plastic, and aluminum. Furthermore, the material maintained its efficiency for aged fingerprints, highlighting its robustness for real-world forensic applications. Although the obtained results demonstrate the effectiveness of the SBA-15/PhCN hybrid for fingerprint visualization, further investigations involving a larger number of forensic samples, different environmental conditions, and more complex substrates are required to fully assess its practical applicability. By avoiding toxic reagents like cyanoacrylate and heavy-metal-based nanoparticles, this hybrid system offers a sustainable, noninvasive, and cost-effective alternative to conventional forensic powders.

## Data Availability

Data will be made available upon request.
